# Unidirectional P-Body Transport during the Yeast Cell Cycle

**DOI:** 10.1371/journal.pone.0099428

**Published:** 2014-06-11

**Authors:** Cecilia Garmendia-Torres, Alexander Skupin, Sean A. Michael, Pekka Ruusuvuori, Nathan J. Kuwada, Didier Falconnet, Gregory A. Cary, Carl Hansen, Paul A. Wiggins, Aimée M. Dudley

**Affiliations:** 1 Institut de Génétique et de Biologie Moléculaire et Cellulaire, Illkirch, France; 2 Luxembourg Centre for Systems Biomedicine, University of Luxembourg, Esch-sur-Alzette, Luxembourg; 3 National Center for Microscopy and Imaging Research, University of California San Diego, La Jolla, California, United States of America; 4 Institute for Systems Biology, Seattle, Washington, United States of America; 5 Tampere University of Technology, Pori, Finland; 6 BioMediTech, University of Tampere, Tampere, Finland; 7 Physics and Bioengineering, University of Washington, Seattle, Washington, United States of America; 8 Centre for High-Throughput Biology, Department of Physics and Astronomy, University of British Columbia, Vancouver, British Columbia, Canada; 9 Molecular and Cellular Biology Program, University of Washington, Seattle, Washington, United States of America; 10 Pacific Northwest Diabetes Research Institute, Seattle, Washington, United States of America; University of Strasbourg, France

## Abstract

P-bodies belong to a large family of RNA granules that are associated with post-transcriptional gene regulation, conserved from yeast to mammals, and influence biological processes ranging from germ cell development to neuronal plasticity. RNA granules can also transport RNAs to specific locations. Germ granules transport maternal RNAs to the embryo, and neuronal granules transport RNAs long distances to the synaptic dendrites. Here we combine microfluidic-based fluorescent microscopy of single cells and automated image analysis to follow p-body dynamics during cell division in yeast. Our results demonstrate that these highly dynamic granules undergo a unidirectional transport from the mother to the daughter cell during mitosis as well as a constrained “hovering” near the bud site half an hour before the bud is observable. Both behaviors are dependent on the Myo4p/She2p RNA transport machinery. Furthermore, single cell analysis of cell size suggests that PBs play an important role in daughter cell growth under nutrient limiting conditions.

## Introduction

RNA granules are aggregates of translationally silenced mRNA and associated proteins that appear as cytoplasmic foci. RNA granules are found throughout the eukaryotic lineage and influence biological processes ranging from germ cell development to neuronal plasticity [1], [2]. Many of these RNA granules transport RNA to specific locations. In *C. elegans* and *Drosophila*, germ granules transport maternal RNAs from the oocyte to the embryo [3]. In mammalian neurons, neuronal granules transport RNA over long distances to the synaptic dendrites where translation can initiate in response to stimulation [4]. Disrupting proper localization of these granules contributes to developmental and neurological diseases [5].

Cytoplasmic processing bodies (p-bodies, PBs) are the most thoroughly characterized RNA granule in yeast. P-bodies contain translationally silenced RNA transcripts and proteins involved in RNA degradation. These protein factors include a cytoplasmic 5′-3′ exonuclease (Kem1p), the catalytic subunit of the mRNA decapping enzyme (Dcp2p), decapping enhancers (such as Dcp1p, Edc3p, Dhh1p, and Pat1p/Lsm1-7p), and proteins involved in nonsense mediated mRNA decay [6], [7]. The RNA molecules contained within p-bodies can be held in a translationally silenced state, degraded, or released back into the translating pool [6], [8], [9].

PBs aggregate rapidly in response to a variety of cellular stresses including glucose depletion, osmotic stress, UV irradiation, acid stress, and DNA damage [9]–[12]. The foci can also disaggregate rapidly once the stress is relieved [13]. The accumulation of PB proteins into cytoplasmic foci does not appear to be a nonspecific aggregation process, but rather a direct response to cell signaling cascades. For example, in yeast PB aggregation in response to high salt is dependent on the function of the effector kinase of the osmotic shock signal transduction pathway (Hog1p) [9]. Despite its thorough characterization, the functional relevance of PB aggregation has yet to be determined. While the protein components of PBs primarily consist of cellular RNA decay machinery, there is no direct evidence that the enzymatic activity of these proteins is altered within PBs [14] or that mRNA decay activities change when PB foci are depleted [15], [16]. There is, however, recent evidence that sequestration of mRNAs into RNA granules may promote cell viability and long term survival under some conditions [17], [18].

To study the spatiotemporal dynamics of p-bodies and their relationship to fundamental cellular processes, we used microfluidic-based fluorescent microscopy and automated image analysis to follow p-body dynamics during yeast cell division in individual cells. Our results demonstrate that under low glucose conditions, these highly dynamic granules undergo a unidirectional transport from the mother to the daughter cell during mitosis. This transport is dependent on the Myo4p/She2p/She3p RNA transport machinery. We also show that p-bodies exhibit a constrained motion near the bud site as much as half an hour before the bud is observable and that this ‘corralled’ movement is also Myo4p and She2p dependent. Furthermore, we find that the presence of p-bodies correlates with cell size, specifically in a *she2*Δ mutant, where daughter cells that receive a p-body grow significantly larger than those that do not. Taken together, these results suggest a role for p-body transport in cell growth and size control under nutrient limiting conditions.

## Materials and Methods

### Yeast Strains and Growth Conditions

Unless noted all yeast media, growth and manipulation is as described [19]. Strains used in this study include: YAD50 (MAT**a** *his3*Δ*1 leu2*Δ*0 met15*Δ*0 ura3*Δ*0 EDC3-GFP::HIS3MX6*) [20], YAD391 (MATα *myo4*Δ*::kanMX4 his3*Δ*1 leu2*Δ*0 lys2*Δ*0 ura3*Δ*0*) [21], YAD421 (MAT**a** *his3*Δ*1 leu2*Δ*0 lys2*Δ*0 ura3*Δ*0 myo4*Δ*::kanMX4 EDC3-GFP::HIS3MX6*) (this study), YAD425 (MATα *she2*Δ*::kanMX4 his3*Δ*1 leu2*Δ*0 lys2*Δ*0 ura3*Δ*0*) [21], YAD426 (MAT**a** *his3*Δ*1 leu2*Δ*0 lys2*Δ*0 ura3*Δ*0 she2*Δ*::kanMX4 EDC3-GFP::HIS3MX6*) (this study), YAD487 (MAT**a** *his3*Δ*1 leu2*Δ*0 met15*Δ*0 ura3*Δ*0 she3*Δ*::kanMX4 EDC3-GFP::HIS3MX6*) (this study). The *she3* deletion was constructed for this study by PCR amplifying the KanMX4 module from a *kem1*Δ strain as in [22] and transforming into YAD50. YAD421 is a haploid segregant of a cross between YAD50 and YAD391. YAD426 is a haploid segregant of a cross between YAD50 and YAD425.

All strains were grown in standard rich medium (YPD, 2% glucose) at 30°C overnight and then diluted in synthetic complete medium (SC, 2% glucose). After reaching exponential growth, cells were concentrated by centrifugation and loaded into the microfluidic device where SC medium containing 2% or 0.1% glucose was circulated as described (Results).

### Microfluidics

#### Device design

**Figure 1:**
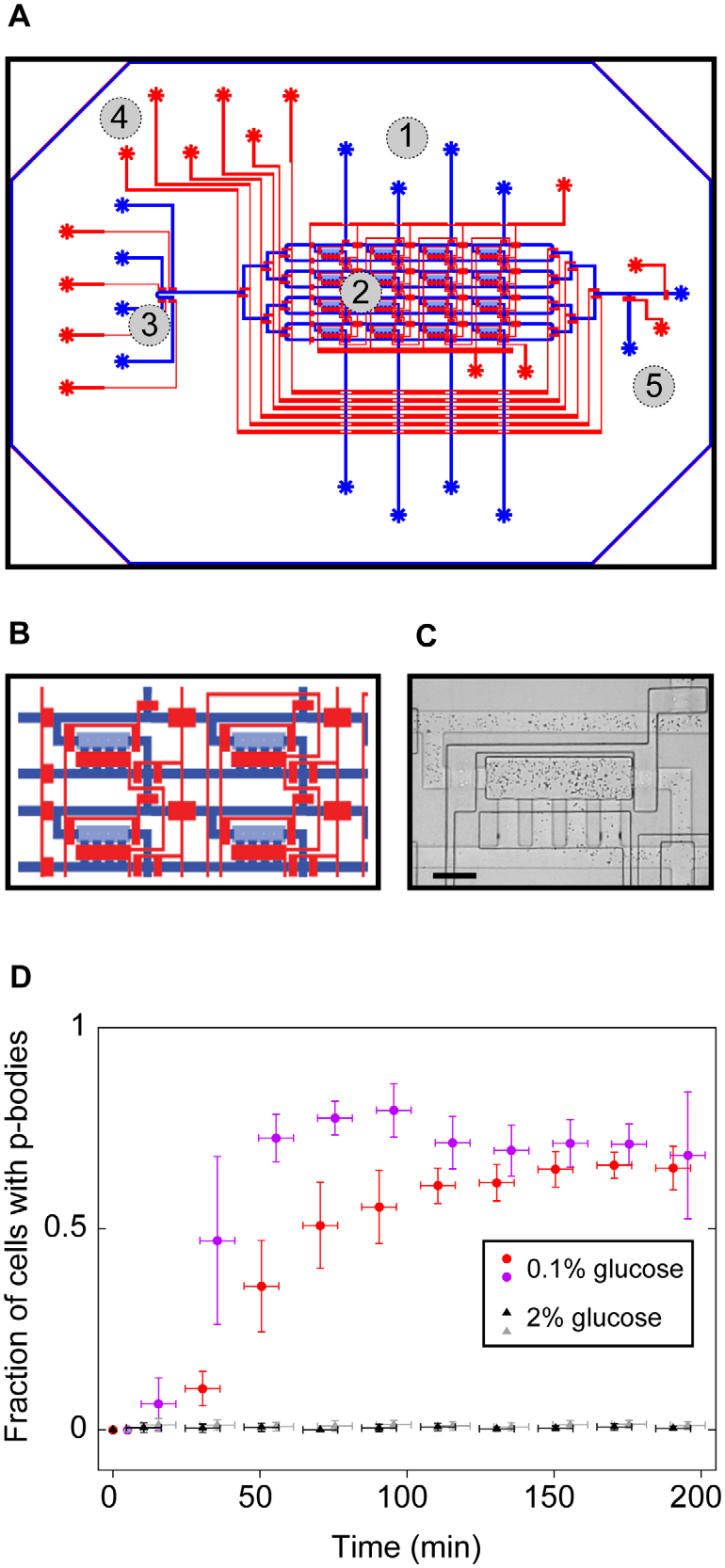
Microfluidics device for studying p-body localization in yeast. (**A**) The device is a simplified version of a published design [23] that consists of 16 chambers in a 4-by-4 matrix with four media inputs accessible via rows and four cell inputs accessible via columns. The device has two layers: the control layer (red) and flow layer (blue). Cells and media are transported within the flow layer channels with the direction controlled by actuating overlaying control layer valves. Parts of the device are marked as follows: 1) cell-loading inlets, 2) chambers where cells are trapped, 3) medium inputs, 4) multiplexer to deliver medium to specific chambers, 5) waste outlet. (**B**) Close up of 4 chambers with the controlling valves. (**C**) Micrograph of a chamber with cells trapped, seen at a 4x magnification in bright field light illumination. Scale bar is 20 µm. (**D**) P-body formation in response to low glucose. Graph shows results from 2 experiment controls (black and grey) in the presence of 2% glucose medium and 2 experiments (purple and red) where p-bodies were induced in the presence of 0.1% glucose medium (4 experiments, total number of cells: 400). Images were taken every 60 seconds over 200 min, in bright field and fluorescent light. The resulting cell numbers were averaged over 20 min periods to filter noise. Custom software for automated quantification of p-bodies per cell was used (see Methods for a detailed description of the analysis).

The P-body 3.0 device is a simplified version of a design from [23]. It consists of 16 chambers in a 4-by-4 matrix with four media inputs accessible via rows and four cell inputs accessible via columns (Fig. 1A). Chamber dimensions are 680 µm by 180 µm allowing a large capture area for cell imaging. The chamber height is set at 3.8±0.1 µm to facilitate cell loading while preventing cell movement during imaging. The flow line height is 9 µm and control lines are 20 µm high.

The cell loading procedure is described in Fig. S1B. Cells are loaded using 3 psi pressure, which causes the chambers to swell enough for cells to flow though. Once the pressure is reduced, cells remain trapped within the chambers due to the constrained height. Media can then be diffused into the chambers by opening the diffusion valve at 1 psi (Fig. S1B and Video S1).

#### Device fabrication

Microfluidic devices were fabricated with PDMS (Momentive Performance Materials - RTV615) by conventional multilayer soft lithography techniques [24]–[26] using two layers and the push-down approach.

#### Device control

Pressure for the control lines was switched between 0 and 30 psi using microsolenoid valves (SMC) and controlled with a custom 32-bit breakout board via NI-cDAQ chassis with NI 9477 module (National Instruments). The software for both manual control and automated switching of media types at specified time points was written in LabVIEW (National Instruments).

### Microscopy

All images were acquired using a motorized Leica DMI 6000B microscope with a Leica HCX PL FLUOTAR, 63x/0.70 corr, air objective and Roper Scientific NTE/CCD-512-EBFT-GR-1 camera (S/N: A040110). Fluorescence illumination was achieved using an Osram HXP-R 120W/45C VIS Mercury short-arc reflector lamp at minimum intensity. The equipment and microfluidics device were kept at constant temperature (30°C) by surrounding the microscope apparatus with an incubation chamber (Leica tempcontrol 37-2 digital). The microscope was placed on a Vibraplane (Kinetic Systems) anti-vibration table. Image acquisition was driven by Metamorph 7.5 software (Molecular Devices). Images were acquired in two different ways. For images acquired at 60 sec intervals over 14 hours, each time point consisted of one image acquired in the fluorescent channel that was complemented by a stack of 40 images (0.3 µm) in transmission light. For images acquired at 10 sec intervals over 14 hours, each time point consisted of only a single image in the fluorescent channel. Fluorescent light images were exposed for 500 ms with the lamp set at 50% of its intensity.

### Image Analysis

Custom software for automated quantification of cells containing p-bodies (Fig. 1D) was developed using MATLAB (Mathworks, Natick, MA). This software detects cells and p-bodies from the bright field and fluorescence images, respectively, and quantifies the number of cells that contain a p-body. First, spots are detected from fluorescence microscopy images using a modification of a band-pass filtering based detection method [27]. The band-pass filtering is implemented as a Difference of Gaussians (DoG), where difference of two low-pass filtered images was taken and the resulting difference image was thresholded into a binary image. The DoG filtering is controlled by setting two Gaussian low pass filters. The filter widths (3 and 9) were determined empirically such that the resulting image contains only the spots, and thus the thresholding could be done with a constant value determined experimentally for each image set. The binary image was further processed by applying size-constraints for potential objects of interest. Then, cells were detected from bright field stacks by determining the differential image of two focus frames [28] and successive application of thresholding and size constraints to remove small and large objects caused by noise, debris and visible background objects, such as device borders. Again, the threshold values were determined empirically such that the cells were preserved in the segmentation result. The detected cells are used as a marker for a standard watershed segmentation [29]. This segmentation result is then used for assigning the detected p-bodies to individual cells, resulting in the fraction of cells with one or more p-bodies.

For further analysis, time frames of single cells over the course of a complete cell division were selected manually. Images were first analyzed manually by tracking p-bodies with the MtrackJ plugin for ImageJ [30]. For the automated high throughput analysis, fluorescent foci were identified using customized MATLAB (Mathworks, Natick, MA) scripts. Briefly, fluorescence microscopy images were analyzed to coarsely isolate individual foci using a watershed-based segmentation algorithm [31], then the position and size of each focus found was determined using a Gaussian point-spread fitting function. False-positives were removed by assigning a score to each focus proportional to the ratio between peak intensity and standard deviation of the fitting function, such that only bright, well-localized foci were kept for analysis.

All image analysis scripts are freely available upon request.

### Data Analysis

To characterize p-body movement, the segmented individual p-body trajectories within single cells were processed using in-house developed software that was implemented in C++ and statistically evaluated using the R environment (version 2.15.0). The image extracted data structure was consistent with the interpretation of movement as a random walk in 2 dimensions. To split data points into separated clusters, we applied the k-means clustering algorithm of the R package (R-clv-0.3_2-1 RPM for x86_64) and correlated the cluster affiliation of each spatial coordinate with its corresponding observation time. The resulting temporal cluster affiliations allows for quantifying transitions between clusters by the reversibility rate. Cluster betweenness, as a measure of how separated the identified clusters are, is defined by J_4_ = tr(S_b_)/tr(S_w_) where S_b_ and S_w_ are the between-class matrix and within-class matrices, respectively [32]. For biophysical interpretation, the extracted trajectories were processed by the in-house developed pipeline with respect to the step size of the considered random walk, velocities and temporal resolved distance of the p-body from the bud. To ensure stationarity, we increased the sample rate from one image per minute to one image per 10 seconds. Step size and velocities were directly calculated from two successive spatial coordinates and not estimated from assumed distributions. To compare p-body movement statistically across different granules of diverse cells and different strains, the mean square displacement (MSD) and related diffusion coefficient [33] were individually calculated and averaged. Statistical significance was tested by Kolmogorov-Smirnov test using R.

All data analysis scripts are freely available upon request.

## Results

### Microfluidics Device to Follow p-body Localization during the Yeast Cell Cycle

Yeast cells growing under optimal conditions have small p-bodies (PBs) that are difficult to detect with standard microscopes. However, PBs rapidly aggregate in response to environmental stresses, such as nutrient deprivation, and rapidly disaggregate once the stress is relieved [9], [13]. Therefore, fluctuating external conditions present a challenge for studying PB dynamics, especially in processes such as the cell cycle that occur over relatively long time scales. To characterize PB behavior in individual cells over multiple cell cycles, we designed a microfluidic, live-cell imaging device that cultures yeast under controlled environmental conditions (Fig. 1A–C) [23]. The device traps cells in chambers whose narrow height limits their growth to a monolayer, thereby allowing the visualization and image analysis of daughter cells growing in the same focal plane as their mother. Medium can flow rapidly into the cell chambers, as demonstrated by a fluorescent dye tracer (Video S1 and Fig. S1A), and cells in the chamber grow with a generation time equivalent to that measured for a large-volume cultures (106 minutes for a wild-type strain in minimal medium (SCD) with 2% glucose). Thus, conditions in the device are conducive to the growth of yeast in a manner that facilitates the imaging of individual cells.

We visualized P-bodies *in vivo* using a known PB component, Edc3p [15] fused to GFP [20]. To study PB movement during the yeast cell cycle, we chose a condition (low glucose) in which PBs were visible, but cells were still able to grow and divide. In 0.1% glucose, PBs formed in most cells after 60 minutes, and cells divided with an average doubling time of 200 minutes. Although the time required for the initial formation of PBs is slower than that observed for complete glucose withdrawal (<10 minutes) in batch culture [9], [13] or microfluidic device (Fig. S2), once formed, PBs were stable as long as conditions were kept constant by circulating the low glucose medium through the device. In contrast, relatively few PBs were observed when the device was infused with the higher glucose concentrations (2% glucose) typically used for batch culture growth (Fig. 1D). These results demonstrate that the formation of PB is neither induced nor inhibited by the microfluidic environment or other conditions of the system (e.g. the fluorescent light), but is instead a specific response to low glucose levels.

### P-body Transport from Mother to Daughter Cell

**Figure 2:**
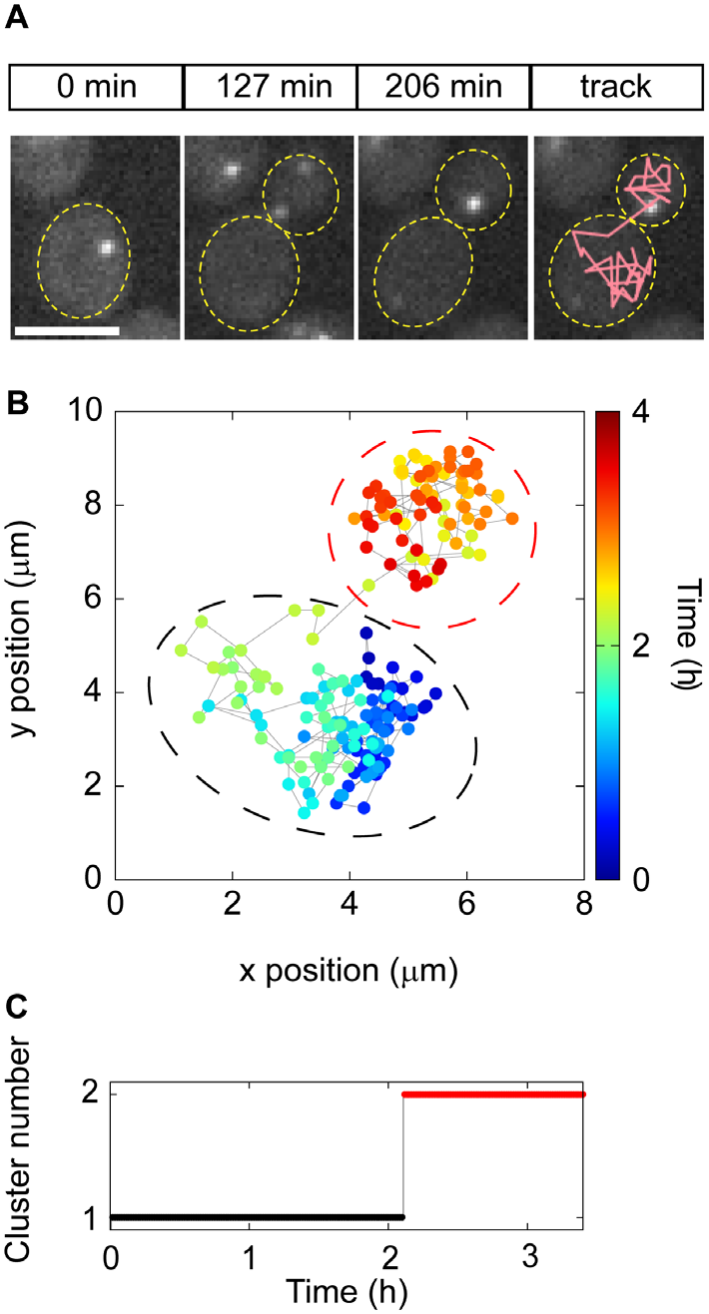
Description of the analysis of p-body dynamics, an example from one cell. (**A**) Time lapse imaging of a p-body during cell division. A wild type strain expressing Edc3-GFP grown in 2% glucose to logarithmic phase was loaded into the microfluidic device. Minimal medium containing 0.1% glucose was flowed for 10 hours and images were acquired every 60 sec in bright field and fluorescent light. A sequence of images spanning 140 min was extracted from the entire experiment. 3 typical images from the time-lapse experiment are shown for the specified time points. In the last panel, the path of the p-body during the cell division is shown. For ease of visualization, the tracks in the microscopy image show PB locations every 5 min. as opposed to every 1 min (the rate of imaging) in Fig. 2B. Scale bar is 5 µm. (**B**) Spatial coordinates of the p-body where color code identifies the observation time. Dashed circles separate the 2 clusters, cluster 1 in black and cluster 2 in red. (**C**) Cluster analysis. Points in (B) where classified in 2 clusters and the corresponding cluster number is shown over time. This clearly demonstrates that a p-body is first localized in one cluster, in this case within the mother cell, (black cluster 1) and is then unidirectional transported to the daughter cell (red cluster 2).

As an initial survey of PB movement during the cell cycle, we grew yeast in low glucose medium and acquired images at 60 second intervals over a 10 hour time course, which typically captured at least three generations of cell division before cell growth and crowding obscured the image analysis. In these experiments, bright field images were used to visualize the cell boundaries and fluorescent light images to visualize PBs. Consistent with observations in mammalian cells [34], PBs in yeast exhibited highly dynamic intracellular movement. However, in contrast to mammalian cells where PBs disassemble during mitosis [35], [36], when yeast were held in low levels of glucose, we observed PBs throughout the cell cycle. Interestingly, in 70% of cells analyzed (n = 61), PBs moved from the mother to daughter cell during cell division in both haploids (Fig. 2A and Video S2, Part I) and diploids (Video S2, Part II), two cell types that exhibit distinct budding patterns due to the activity of different sets of bud-site selection proteins [37]. Finally, although most cells contained a single PB, when cells contained multiple PBs, all PBs usually moved to the daughter cell. These results suggested that PBs may be specifically transported from mother to daughter during cell division.

### Unsupervised Method to Analyze p-body Dynamics

To objectively quantify the properties of these dynamics across a large number of cells and experiments, we developed a series of computational scoring metrics that utilized data derived from our automated image analysis methods. To examine directionality, we developed an unsupervised method to analyze the spatial dynamics of PB movement during one cell cycle. Applying k-means clustering (k = 2) to PB positional (x–y) coordinates identified clusters corresponding to locations in the mother and the daughter cells without prior knowledge of PB movement (Fig. 2B). Consistent with the observation that PBs move from mother to daughter, the two clusters of spatial coordinates correlated with their temporal occurrence, i.e. the earlier time points belonged to one cluster that resided in the mother cell (Fig. 2B, black circle), and the later time points belonged to the second cluster that resided in the daughter cell (Fig. 2B, red circle). To quantify the reversibility of this movement, we examined the temporal behavior of cluster switching (Fig. 2C). In this analysis, a PB that moves from mother to daughter irreversibly will show a single transition, while a PB that reversibly travels between mother and daughter will show multiple transitions. In the majority of wild-type yeast cells, we observed a single transition between clusters, a result that supports a unidirectional transport hypothesis. Taken together, these tools provide the foundation for an objective, unsupervised method of assessing the movement of cytoplasmic foci from a relatively large number of time lapse images.

### P-body Movement is Dependent on the RNA Transport Machinery

With these methods in place, we sought to identify some of the molecular mechanisms underlying these behaviors. In yeast, a protein complex consisting of a myosin (Myo4p), a protein adapter (She3p) and an RNA binding protein (She2p) is responsible for localizing at least 22 RNA transcripts to daughter cells during mitosis [38]. The best characterized of these is the ASH1 mRNA, which is transported by the protein complex along the actin cytoskeleton from the mother to the daughter cell, where the Ash1 protein is synthesized [39].

**Figure 3:**
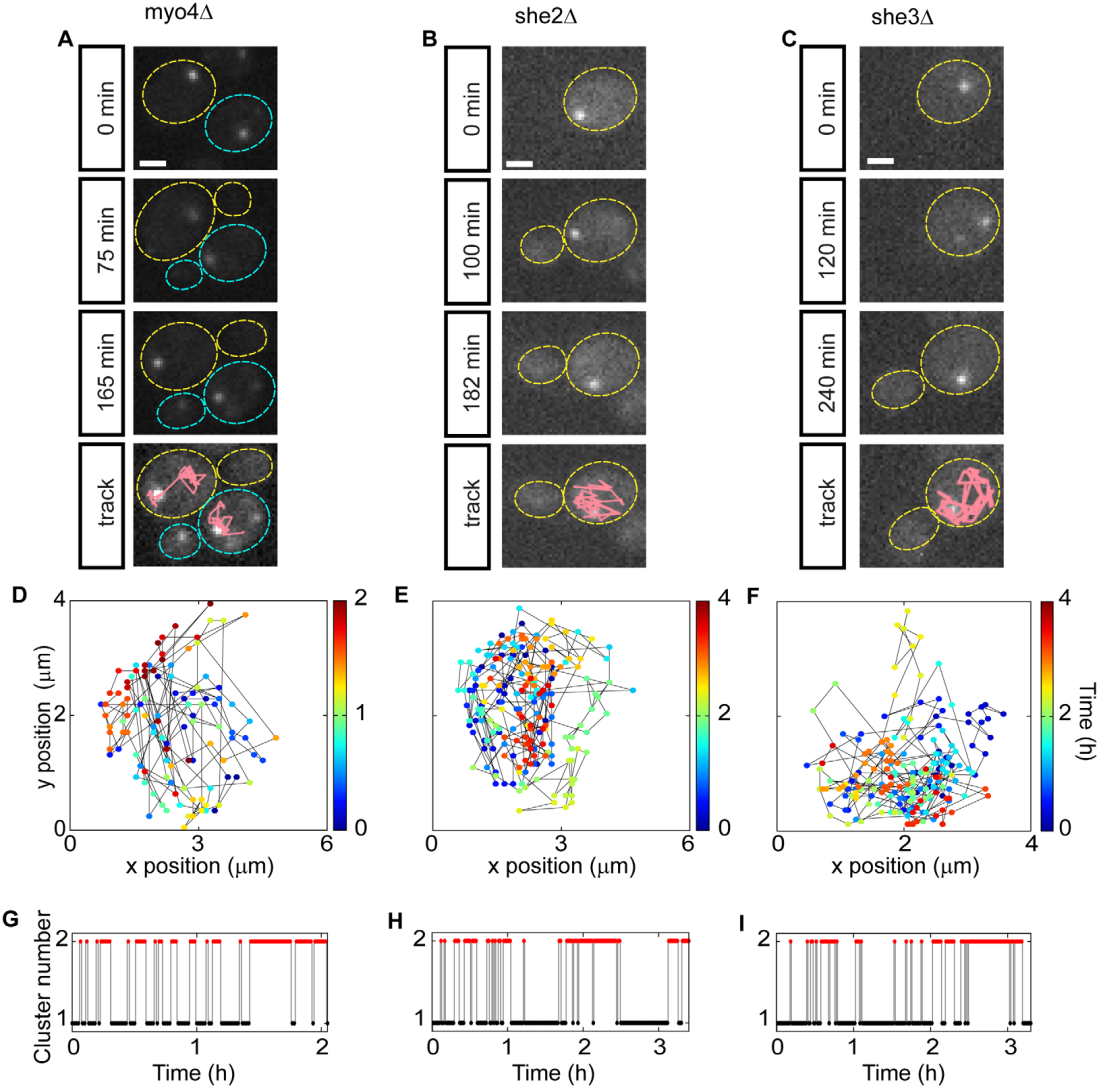
P-body transport to the daughter cell is dependent on Myo4p, She2p and She3p. (**A–C**) Sequence of images tracking p-bodies in strains lacking Myo4p, She2p or She3p. Experiments were performed and plotted as in Fig. 2. One cell is shown here for the *she2*Δ and *she3*Δ strains and two cells are shown for the *myo4*Δ strain. 3 images from the time-lapse experiment are shown. The last panel summarizes the path of the p-body during cell division. Scale bar, 2 µm. (**D–F**) Spatial coordinates of p-bodies from cells shown in (**A**) where color corresponds to time. (**G–I**) Cluster analysis performed in each cell, as in Fig. 2C. In the mutants p-bodies do not move to the daughter cell and p-bodies change from cluster 1 to cluster 2 multiple times.

To test whether this RNA transport complex plays a role in PB movement, we analyzed strains harboring deletions of each of these genes. Analysis of Edc3p-GFP localization in a strain lacking Myo4p showed a clear difference from wild-type cells (Fig. 3A and Video S3, Part I). In a *myo4* deletion mutant (*myo4*Δ), PBs still showed highly dynamic movement within the mother cell. However, in the majority of *myo4*Δ cells (96%, n = 77), PBs failed to translocate to the daughter cell (Fig. 3A), and in some of these cases where the original PB remained in the mother cell, a new PB appeared to form *de novo* in the daughter (Video S3, Part I). Because PBs that remained in the mother cell continued to move dynamically, k-means clusters of the PB spatial coordinates correlated poorly with their temporal occurrence (Fig. 3D). Furthermore, because the clusters represented arbitrary separations of positions within the mother cell that the PB traversed in the course of its dynamic movement, the *myo4*Δ strain also displayed a relatively large number of between cluster transitions (Fig. 3G). A similar effect was seen in the *she3*Δ strain (Fig. 3C, F and I, Video S3, Part II), where PBs remained in the mother cell in 95.4% of cells (n = 65). The *she2*Δ strain showed a significant, but slightly less penetrant effect (Fig. 3B, E and H, Video S3, Part III), with PBs failing to localize to the daughter in 81% of cells (n = 63), including one rare case in which the PB moved back and forth between daughter and mother cell. Together, these results show a strong dependence of mother to daughter PB movement on both Myo4p and She3p, with a weaker effect of She2p.

**Figure 4:**
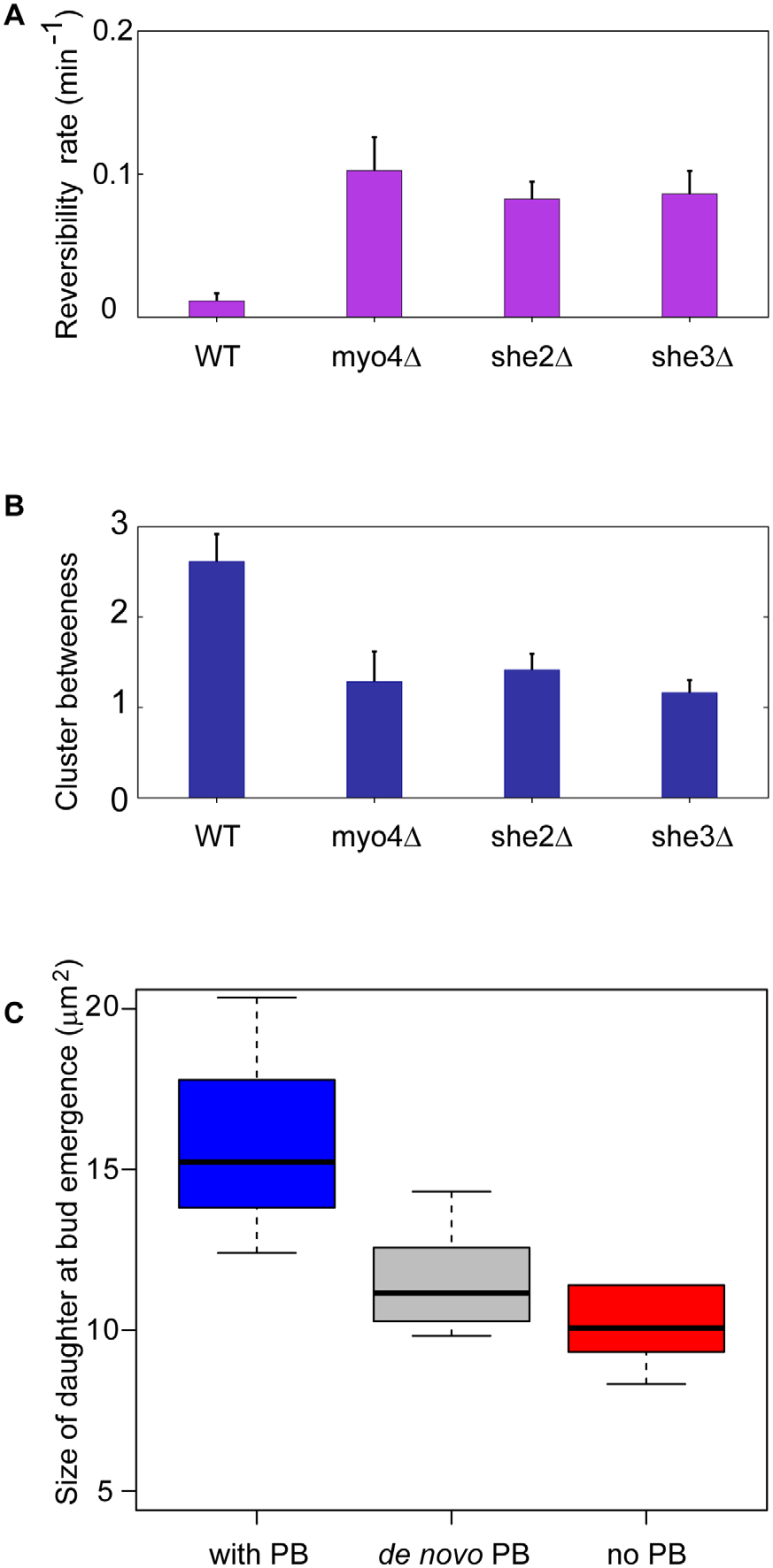
P-body movement is directional and correlates with cell size. (**A**) Reversibility analysis of the movement. A reversibility rate of PB movement was calculated for each strain (wild-type, *she2*Δ, *she3*Δ and *myo4*Δ) for 11, 11, 12 and 16 cells respectively, as explained in the main text. Non-zero values for wild-type typically occur due to a few ‘miss-clustered’ points during the transition phase. (**B**) Cluster betweenness. By relating points within a cluster to points within the other cluster we can quantitatively compare the separation of PB movement between the strains (Materials and Methods). Similar to the temporal analysis by *R_rev_* we see a significant difference between the wild-type and mutant strains. (**C**) Area of she2Δ cells at the time of budding that had received a PB (blue), not received a PB but later formed one *de novo* (gray), and completely lacked a detectable PB (red). The population of cells that received a PB during cell division were 33% larger than cells that did not (p<0.03).

To compare the temporal aspect of PB dynamics across time courses acquired at different temporal resolutions and cells with different cell cycle lengths, we defined a reversibility rate *R*_rev_ that relates the number of cluster transitions (τ) to the number of time points sampled (*n*) for each cell:
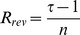



*R_rev_* equals 0 for PBs that move irreversibly from mother to daughter cell and converges to 1 for particles that move back and forth between the two cells. Applying this measure to our full set of experiments, we observed a clear and statistically significant difference between the wild-type and mutant strains (Fig. 4A), with wild-type cells showing a significantly lower reversibility rate. To objectively assess the spatial properties of PB movement in a large number of cells, we calculated the cluster betweenness [32] (Fig. 4B), which measures the separation of clusters by comparing the proximity of points within a cluster to points in a different cluster. By this metric, PBs that move to the daughter cells should have a larger cluster distance than PBs that stay in the mother (Fig. S3). Results from this analysis showed significantly (p<0.001) larger cluster distances for wild-type cells compared to the mutant strains (Fig. 4B). These results demonstrate a statistically significant difference between the movement of PBs in wild-type cells and strains lacking components of the RNA transport machinery.

### P-bodies Influence Daughter Cell Growth

Having characterized this behavior and some of the cellular machinery required for it, we next sought to use this system to test whether PB transport had a physiologically relevant effect on either mother or daughter cells. In our experiments, daughter cells could be divided into three classes (Table 1) with respect to their PB status: 1) those that received a PB from the mother, 2) those that did not receive a PB but generated one *de novo* following cell division, and 3) those that completely lacked a detectable PB. We quantified several parameters of individual mother and daughter cells in which we could track PB localization and confidently assign cells to these categories. To control for heterogeneity that might be caused by differences in cells acclimating to the environment, we examined only cells born in the microfluidic chip under the low glucose condition. To obtain reliable statistics, we took advantage of the fact that the s*he2*Δ strain produces sufficient numbers of daughter cells in all three classes (Table 1). While most of the parameters we examined showed no significant difference between these classes, we detected a striking difference in daughter cell size that correlated with whether or not the cell had received a PB. More specifically, by the time these daughter cells began to bud, cells that had received a PB during cell division had become 33% larger than cells that had not (p<0.03, Fig. 4C). Daughter cells that appeared to form a PB *de novo* were also smaller than cells that had inherited a PB (Fig. 4C). Finally, despite the low frequency of such events, we also saw a significant size difference between wild-type daughter cells that lacked a detectable PB and the other two classes that contained a PB as a result of either transport or *de novo* formation (Fig. S4). We note that the apparent differences between the wild-type and *she2*Δ strains for the *de novo* formation class may either be a result of the small numbers of cells obtained for some classes or may reflect some underlying function of the She2 protein. Together, these results strongly suggest that the presence of PBs plays an important role in daughter cell growth under nutrient limiting conditions.

**Table 1:** P-body localization classes in wild-type, *myo4*Δ, and *she2*Δ cells (for n = 42, 48, 44 p-bodies).

*Strain*	*PB transported from mother*	*PB formed de novo*	*No PB detected*
**wild-type**	70%	26%	4%
**myo4Δ**	4%	64%	32%
**she2Δ**	19%	44.5%	36.5%

### P-body Hovers near the Bud Site Prior to Bud Emergence

**Figure 5:**
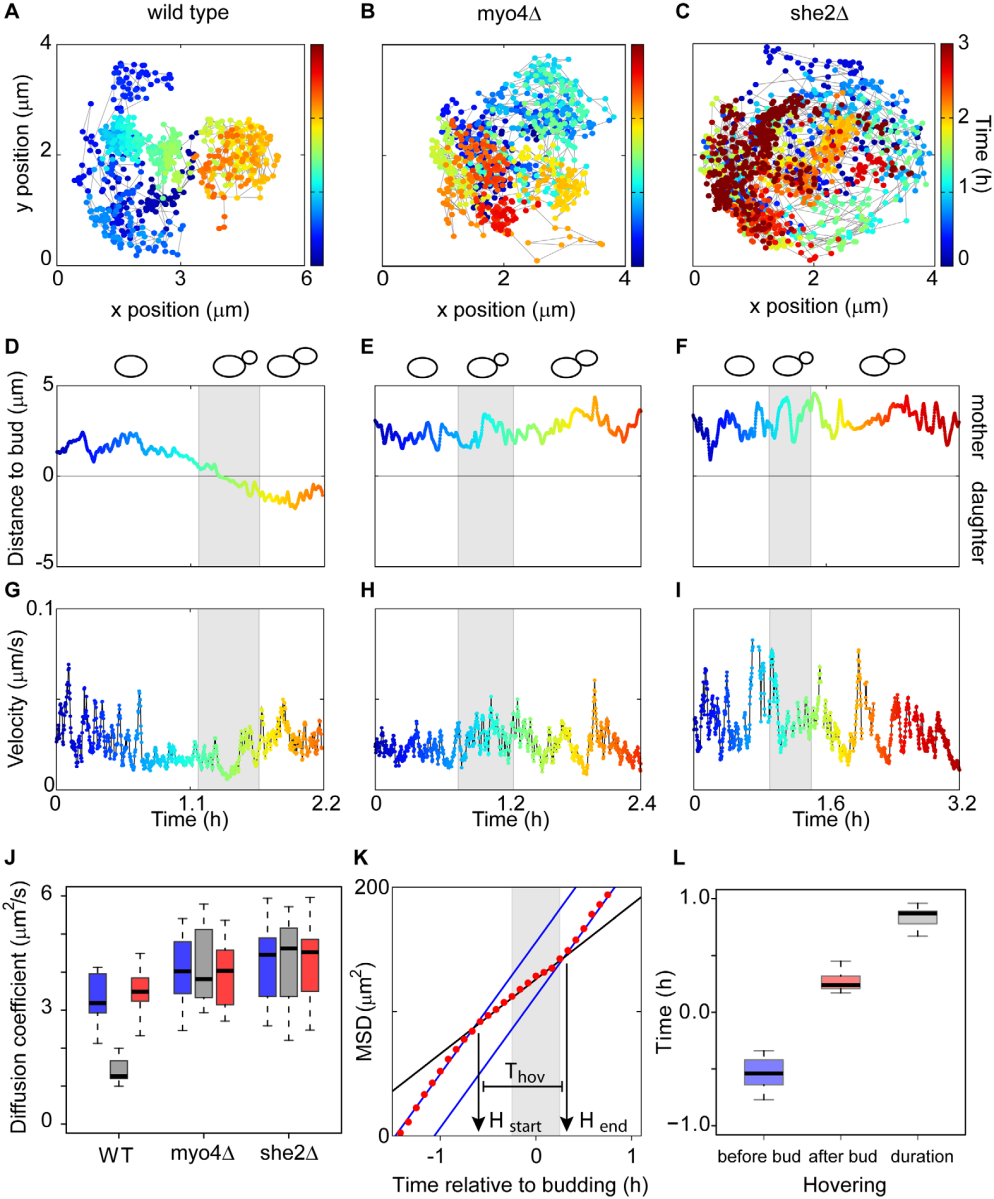
Hovering of PB close to the bud site in a Myo4p and She2p dependent manner. (**A–C**) Spatial coordinates of PBs in wild-type, *myo4*Δ and *she2*Δ cells from images acquired every 10 seconds. The x and y axis correspond to image coordinates. Time is color coded with the same scale for the 3 panels. (**D–F**) Bud to PB distance during the cell cycle in wild-type, *myo4*Δ, and *she2*Δ cells. 0 marks the bud site (manually located). Budding time (15 min before and 15 after bud formation) is indicated in grey. A scheme of cell cycle progression is drawn above the plots. (**G–I**) Velocity of PB over time. The grey region is as in (D–F). (**J**) Diffusion coefficients before (blue), during (grey) and after (red) budding for the wild-type, *myo4*Δ and *she2*Δ strains. (**K**) Mean square displacement (MSD) for the wild-type cell shown in (A). Blue lines describe diffusion before and after budding; the black line describes the diffusion during bud formation. H_start_ is the beginning of hovering. H_stop_ is the end of hovering. T_hov_ is total time of hovering. 0 corresponds to the start of budding. (**L**) Analysis of the hovering time in 11 wild type cells. The H_start_ average is 0.55 hours before budding, H_stop_ average is 0.27 hours after budding and the total time of hovering, T_hov_ in average is 0.83 hours.

In addition to mother-daughter transport, visual inspection of time lapse images suggested another PB behavior that correlated with bud formation. To characterize PB movement in more detail during a specific phase of the cell cycle, we increased the frequency with which cells were imaged to every 10 seconds over the 10 hour time course, a speed that limited image acquisition to a single type of illumination (fluorescent light for the detection of GFP). Analysis of this higher resolution time course data confirmed the unidirectional and myosin dependent transport results (Fig. 5A–C and Fig. S3). The fine-scale time points also permitted a random walk based analysis of the distance from the PB to the bud site in wild-type and mutant cells (Fig. 5D–F). Interestingly, in wild-type cells, PBs appeared to localize near the future bud site several minutes before the bud was visible, an event that occurs at the beginning of the S phase of the cell cycle. To quantify this hovering behavior, we analyzed the velocity of PBs over the course of the cell cycle (Fig. 5G–I). In wild-type cells, we observed a significant decrease in PB velocity during bud formation (Fig. 5G, grey shadowed) and even tens of minutes prior to bud emergence. Because the Myo4p/She2p/She3p complex is required for the localization of specific RNA transcripts to the bud [38], [40], [41], we measured PB velocity in *myo4*Δ and *she2*Δ mutants (Fig. 5H–I). Interestingly, these mutants continued to exhibit the dynamic movement of PBs inside the mother cell, but did not exhibit reduced velocities before or during bud emergence. This observation is consistent with a model in which reduced PB velocity depends on interactions with other cellular structures, leading to a “corralled” movement in wild-type cells, where they seem to hover close to the bud site for a significant portion of the cell cycle.

To characterize this hovering behavior in a more quantitative manner, we calculated the diffusion coefficient of individual PBs for defined windows before (blue), during (grey) and after (red) budding in wild-type and mutant cells (Fig. 5J). This strategy facilitated a comparative analysis across strains on a population level and a characterization of PB localization in relation to the cell cycle. Interestingly, wild-type and mutant strains had similar PB diffusion coefficients during the periods before and after budding. During these phases, they adopted a free diffusion regime, as determined by the diffusion like velocity distributions of the granules (Fig. S5). However, during the window that encompasses budding, only wild-type cells showed a significant (p<0.001) reduction of more than 50% in the diffusion strength. This failure to “hover” at the bud site in the mutants suggests that the process is dependent on some function of the Myo4p/She2p RNA localization machinery.

To examine the hovering of PBs and its relation to budding in more detail, we analyzed the temporal behavior of the mean square displacement (MSD), which measures the area that a PB covers during the window of observation. In wild-type cells, we identified two diffusion regimes based on different slopes of the temporal MSD relationship (Fig. 5K). The transition between these MSD values allows the determination of the start (H_start_) and end (H_end_) points of hovering in relation to the budding process. Applying this analysis to individual PBs in different cells from independent experiments demonstrated that PBs exhibit a corralled movement beginning half an hour before the bud is visible and remain in this hovering state for approximately one hour. After the PB is transported to the daughter cell, it resumes diffusion characteristics similar to those observed in the mother cell prior to hovering (Fig. 5L).

## Discussion

Using a combination of microfluidics, fluorescence microscopy, and automated image analysis, we have shown that under at least one condition (0.1% glucose) yeast PBs are present throughout the cell cycle. Overall, this analysis revealed that in wild-type cells PBs exhibit three different classes of movement. The first class is free diffusion inside the mother cell. This movement is independent of the Myo4p/She2p/She3p RNA transport machinery and is not associated with the bud site. The second class is a corralled movement that occurs when PBs hover close to the bud site and is characterized by a Myo4p- or She2p-dependent reduction in PB velocity. This hovering behavior begins approximately 30 minutes prior to bud emergence, during the G1 phase of the cell cycle, and lasts for approximately one hour. The third class is the unidirectional transport of PB from mother to daughter cells by a mechanism that depends on the function of Myo4p, She2p and She3p.

Interactions between PBs and this mRNA localization complex could be direct or mediated by additional factors and the strong effect of deleting the RNA binding protein, She2p, suggests a potential role of the RNAs present in PBs. These results are consistent with observations in other systems in which myosins from the same family are known to tether cellular components to specific subcellular locations, e.g. the human Myosin Va which helps anchor secretory granules to the plasma membrane [42], [43]. Our study has also uncovered a cell cycle dependent transport of an RNA granule to a specific subcellular location, the future site of bud emergence. These results strongly suggest storage and transport functions for PBs in yeast.

Cell size is among the most fundamental of physiological traits. The accurate coordination of cell growth and cell division is important for survival in environments with changing nutrient levels. In budding yeast, control of size is primarily carried out by regulating division in response to growth [44]. This requires the cell to detect nutrient concentrations, growth rates, or cell size. Despite extensive characterization of the mechanisms driving the cell cycle, the means by which cells coordinate their size and cell division is still not well understood. We have shown that (at least under this nutrient limited condition) failure to receive a PB during cell division has significant (30% reduction in growth during the subsequent G1 phase of its cell cycle), but not fatal, consequences for the newly formed daughter cell. While these results are consistent with previous results showing decreased growth rates in nutrient limited conditions for strains with reduced levels of *MYO4*, *SHE2* and *SHE3* [45], by comparing the size of cells that did or did not receive a PB in a common (*she2*Δ) mutant background, we are able to control for other effects which the mutation may cause. Interestingly, daughter cells that did not receive a PB often appeared to assemble a new PB *de novo* (Fig. 3A). The fact that these daughter cells were also significantly smaller than those that had received a PB from the mother (at least in a *she2*Δ strain) suggests that mother to daughter transport of either some specific cargo (presumably specific proteins or RNAs) or sufficient quantities of such cargo present in the large RNA-protein complexes participate in the growth control of the newly formed cells.

This study provides an example of how extending traditional approaches with automated time-lapse imaging of individual cells can identify novel gene functions and spatiotemporal orchestration of elementary cellular processes [46]. While a number of recently described methods for single cell analysis focus on static cell state determination [47], [48], we demonstrate here that combining current microfluidics methods, fluorescent imaging, high-throughput image and in depth computational analysis is able to reveal spatiotemporal dynamics of subcellular structures and suggest their physiological relevance. Analytical strategies similar to those employed here are not limited to the study of RNA granules, but might also be adapted to the study of other cellular aggregates, such as prions. Overall, these results highlight the urgent need for a mechanistic understanding of subcellular dynamics in time and space to decipher cellular regulation in health and disease [49].

## Supporting Information

Figure S1 **A** **Fluorescent tracer flow inside the microfluidics chamber.** A solution of fluorescein (1 mg/ml) was flowed through the microfluidics chamber and images of one chamber were acquired every second. Fluorescence was measured over time and the fluorescence mean of the area (104 pixels^2^) is plotted. **B. Cell loading procedure.** The different combination of open/close valves to (**1**) load cells, (**2**) wash excess to waste, and (**3**) flow medium for one chamber of the device. White arrows represent the flow of cells or medium. Flow lines are colored blue and control lines are colored red. Cells were loaded using 3 psi pressure which causes the chambers to swell enough for cells to flow though. Once the pressure is reduced, cells remain trapped within the chambers due to the constrained height. Media can then be diffused into the chambers by opening the diffusion valve at 1 psi (Video S1).
(TIF)

Figure S2 **P-body formation after glucose removal.** Percentage of cells with visible P-bodies after transitioning from glucose containing medium to medium without glucose. Cells expressing Edc3-GFP were loaded in a microfluidic chamber and images were taken in fluorescent light every 20 seconds over 10 min. Custom software for automated quantification of cells with p-bodies was used (see Methods for a detailed description of the analysis).
(TIF)

Figure S3 **P-body movement.** **A–C**: Spatial coordinates of p-bodies in (**A**) wild-type, (**B**) *myo4*Δ and (**C**) *she2*Δ from images acquired every 10 seconds. X and y axis correspond to image coordinates. Time is color coded. **D–F**: Cluster analysis for each cell from A–C as in Figures 2 and 3. **G**: Reversibility rate in pink for wild-type cells, myo4Δ cells, *she2*Δ cells and from simulated Brownian motion and their respectively cluster betweenness in purple. Data is from the high resolution imaging experiments as described in the main text and Figure 3.
(TIF)

Figure S4 **Area of wild-type daughter cells depending on PB status.** Area of cells that had received a PB (blue), had not received a PB but later formed one *de novo* (gray), and completely lacked a detectable PB (red). Area was calculated immediately prior to the emergence of the first bud from these daughter cells (as a measure of the maximum growth of that cell). The population of cells that did not received a PB during cell division was smaller than cells that did received a PB (p = 0.029) or formed a PB *de novo* (p = 0.068).
(TIF)

Figure S5 **Frequency of velocities.** Frequency of velocities shown in figure 4 D–F in **(A)** a wild type cell, **(B)** a *myo4*Δ cell and **(C)** a *she2*Δ cell. Velocities during 30 min before budding are shown in the upper panel in blue. Velocities during budding are shown in grey in the middle panel and velocities after budding are shown in red in the lower panel.
(TIF)

Video S1 **Fluorescent tracer flow inside the microfluidics chamber.** A solution of fluorescein (1 mg/ml) was flowed in the microfluidics chamber and images of one chamber were acquired every second during 60 seconds.
(MP4)

Video S2 **P-body dynamics in haploid and diploid wild type cells.** Part I: P-body movement in a haploid cell: Images of a wild-type haploid strain expressing Edc3-GFP acquired every 60 seconds during 14 hours. Part II: P-body movement in a diploid cell. Images of a wild-type diploid strain expressing Edc3-GFP acquired every 60 seconds during 14 hours. Bright field images overlap fluorescent images. Several cell divisions can be observed.
(MP4)

Video S3 **P-body dynamics in RNA transport machinery mutants.** Part I: P-body movement in a *myo4* deletion strain. Images of a *myo4*Δ haploid strain expressing Edc3-GFP acquired every 60 seconds during 161 min. Images in Fig. 3A were extracted from this series. Part II: P-body movement in a *she3* deletion strain. Images of a *she3*Δ haploid strain expressing Edc3-GFP acquired every 60 seconds during 217 min. Images in Fig. 3B were extracted from this series. Part III: P-body movement in a *she2* deletion strain. Images of a *she2*Δ haploid strain expressing Edc3-GFP acquired every 60 seconds during 253 min. Images in Fig. 3C were extracted from this series.
(MP4)
