# Supplementary figures and images for: Unidirectional P-Body Transport during the Yeast Cell Cycle
Source: PLoS One. 2014 Jun 11;9(6):e99428. doi: 10.1371/journal.pone.0099428 (PMC4053424; doi:10.1371/journal.pone.0099428)

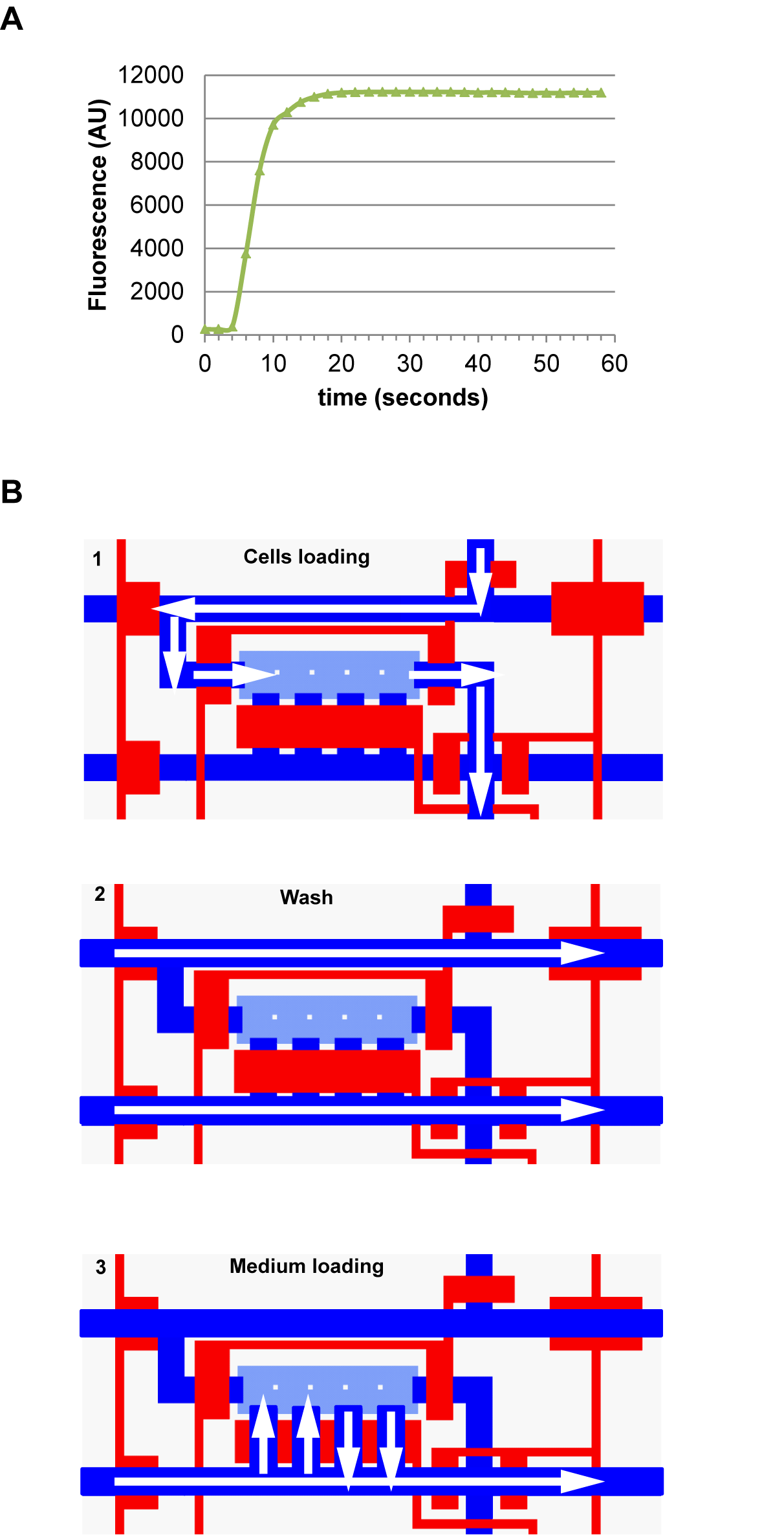

Supplement: Figure S1 — A Fluorescent tracer flow inside the microfluidics chamber. A solution of fluorescein (1 mg/ml) was flowed through the microfluidics chamber and images of one chamber were acquired every second. Fluorescence was measured over time and the fluorescence mean of the area (104 pixels2) is plotted. B. Cell loading procedure. The different combination of open/close valves to (1) load cells, (2) wash excess to waste, and (3) flow medium for one chamber of the device. White arrows represent the flow of cells or medium. Flow lines are colored blue and control lines are colored red. Cells were loaded using 3 psi pressure which causes the chambers to swell enough for cells to flow though. Once the pressure is reduced, cells remain trapped within the chambers due to the constrained height. Media can then be diffused into the chambers by opening the diffusion valve at 1 psi (Video S1). (TIF) [file pone.0099428.s001.tif]

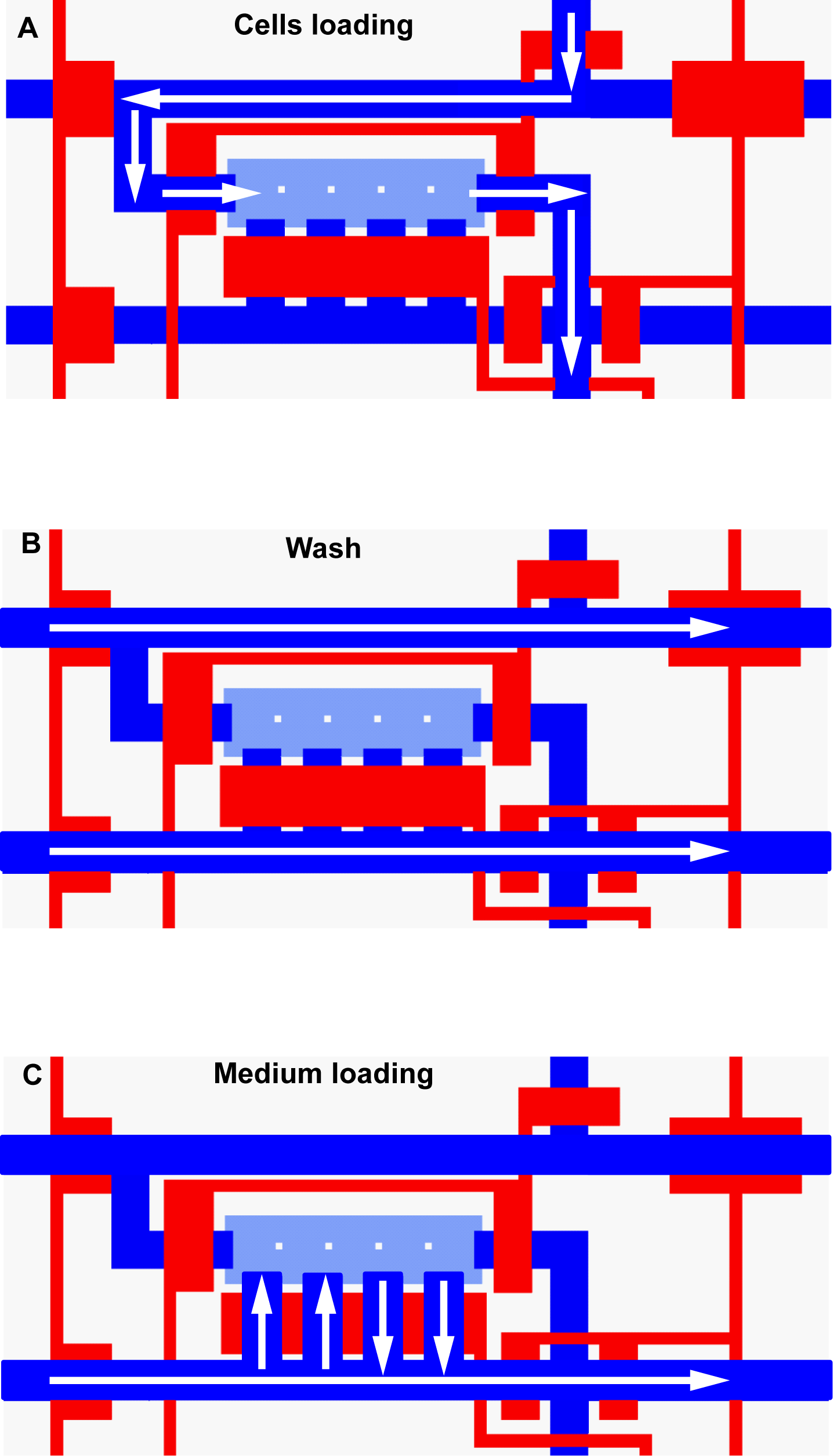

Supplement: Figure S2 — P-body formation after glucose removal. Percentage of cells with visible P-bodies after transitioning from glucose containing medium to medium without glucose. Cells expressing Edc3-GFP were loaded in a microfluidic chamber and images were taken in fluorescent light every 20 seconds over 10 min. Custom software for automated quantification of cells with p-bodies was used (see Methods for a detailed description of the analysis). (TIF) [file pone.0099428.s002.tif]

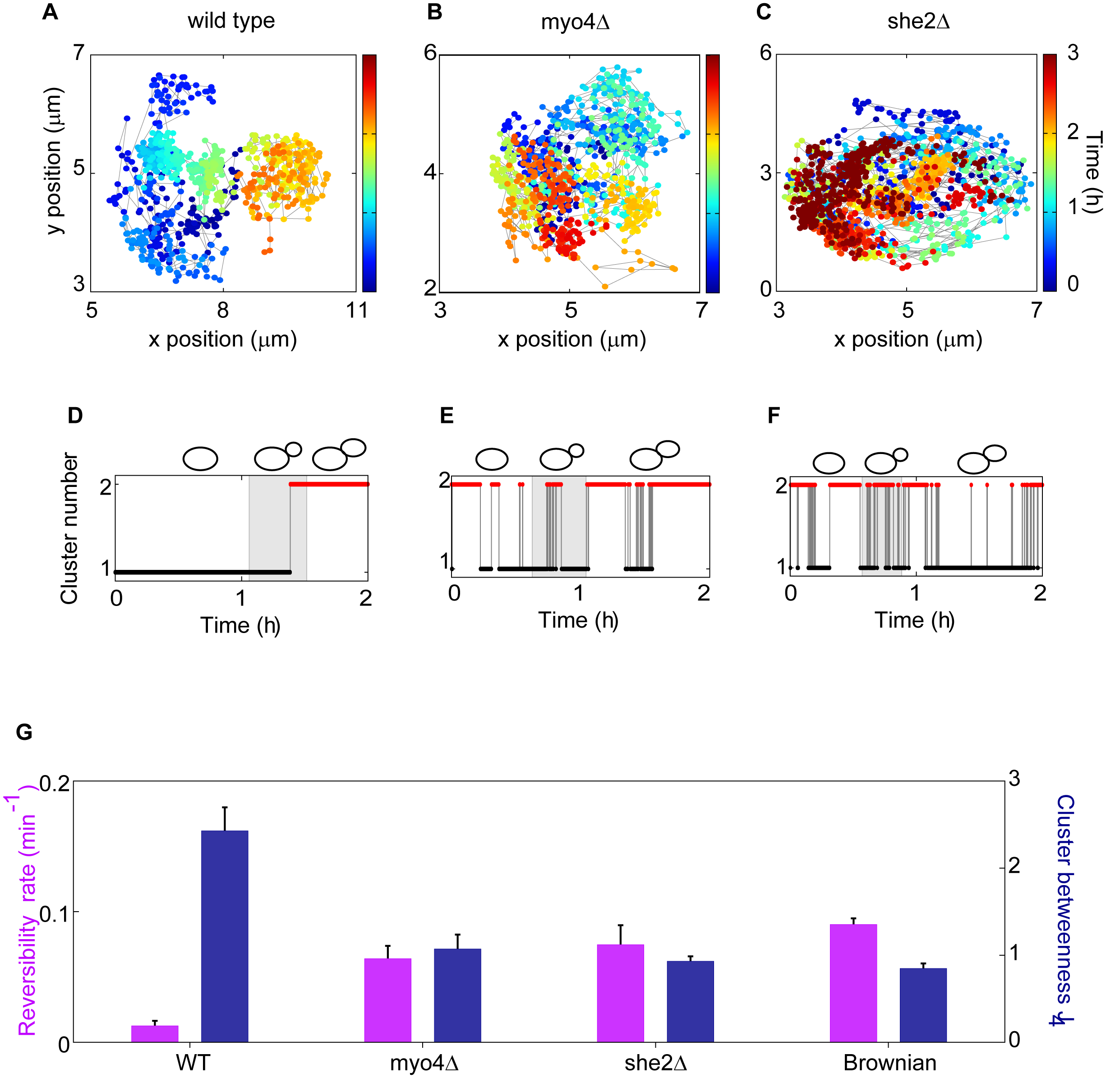

Supplement: Figure S3 — P-body movement. A–C: Spatial coordinates of p-bodies in (A) wild-type, (B) myo4Δ and (C) she2Δ from images acquired every 10 seconds. X and y axis correspond to image coordinates. Time is color coded. D–F: Cluster analysis for each cell from A–C as in Figures 2 and 3. G: Reversibility rate in pink for wild-type cells, myo4Δ cells, she2Δ cells and from simulated Brownian motion and their respectively cluster betweenness in purple. Data is from the high resolution imaging experiments as described in the main text and Figure 3. (TIF) [file pone.0099428.s003.tif]

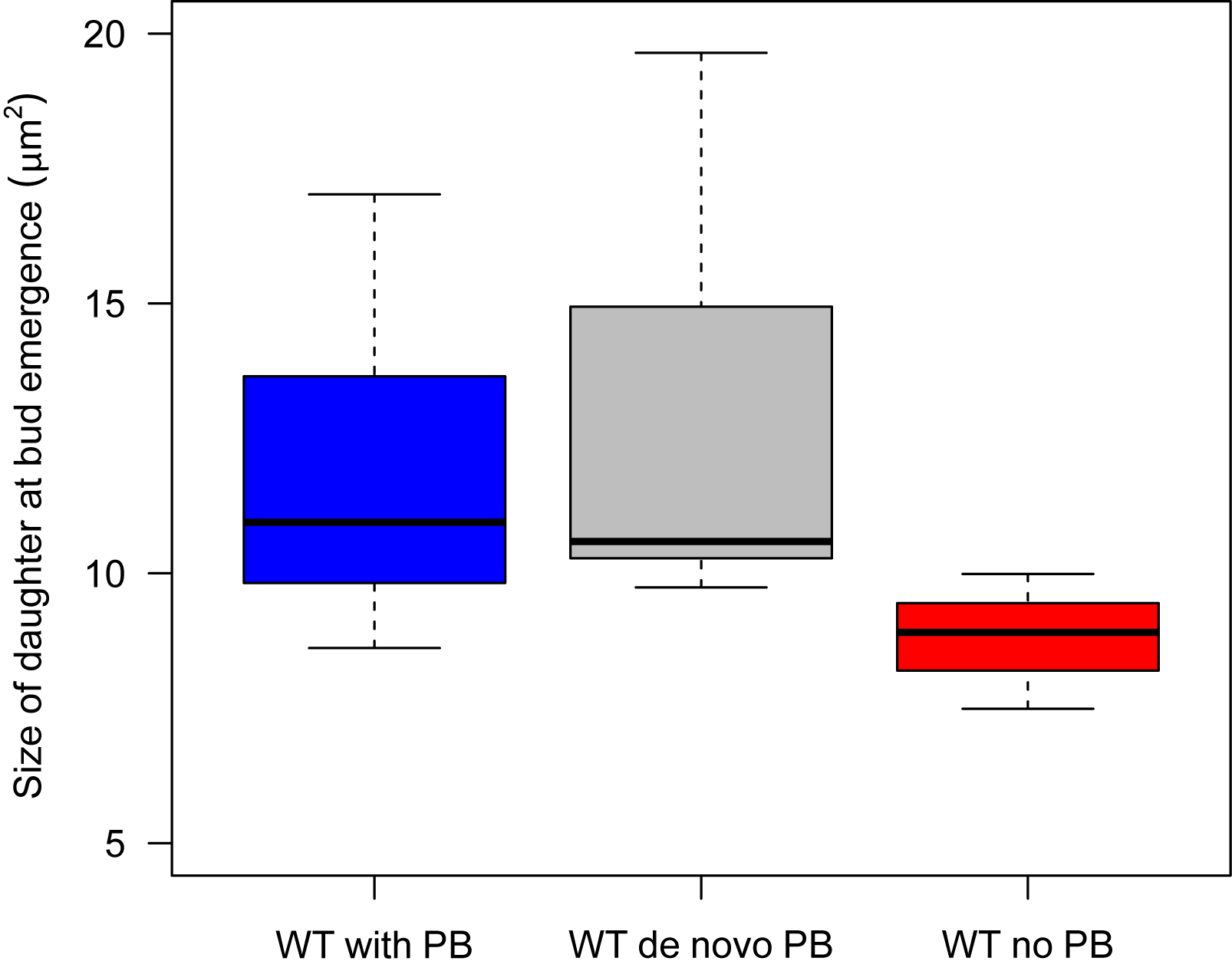

Supplement: Figure S4 — Area of wild-type daughter cells depending on PB status. Area of cells that had received a PB (blue), had not received a PB but later formed one de novo (gray), and completely lacked a detectable PB (red). Area was calculated immediately prior to the emergence of the first bud from these daughter cells (as a measure of the maximum growth of that cell). The population of cells that did not received a PB during cell division was smaller than cells that did received a PB (p = 0.029) or formed a PB de novo (p = 0.068). (TIF) [file pone.0099428.s004.tif]

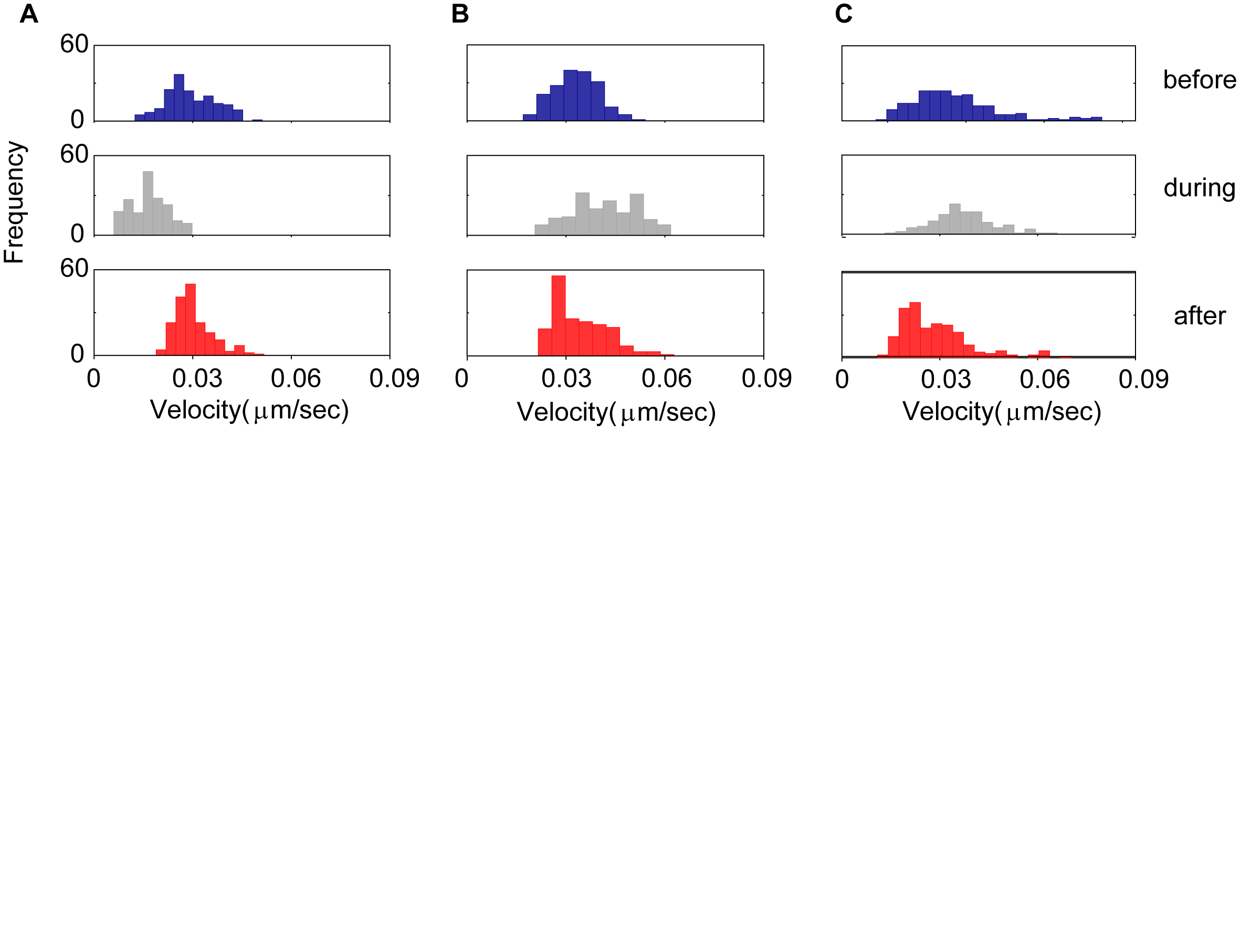

Supplement: Figure S5 — Frequency of velocities. Frequency of velocities shown in figure 4 D–F in (A) a wild type cell, (B) a myo4Δ cell and (C) a she2Δ cell. Velocities during 30 min before budding are shown in the upper panel in blue. Velocities during budding are shown in grey in the middle panel and velocities after budding are shown in red in the lower panel. (TIF) [file pone.0099428.s005.tif]
